# Promising Thermoelectric Performance of Janus Monolayer ZrBrI

**DOI:** 10.3390/ma19091716

**Published:** 2026-04-23

**Authors:** Jingfeng Wang, Wenyan Jiao, Zihe Li, Huijun Liu

**Affiliations:** Key Laboratory of Artificial Micro- and Nano-Structures of Ministry of Education and School of Physics and Technology, Wuhan University, Wuhan 430072, China; wangjf@whu.edu.cn (J.W.); jiaowy@whu.edu.cn (W.J.); lzhlzh@whu.edu.cn (Z.L.)

**Keywords:** Janus monolayer, first-principles, Boltzmann transport theory, thermoelectric properties

## Abstract

The Janus monolayers have recently attracted substantial interest due to their unique asymmetric structures and intriguing physical properties. In this work, we explore the thermoelectric properties of the Janus monolayer ZrBrI, using first-principles calculations and Boltzmann transport theory. We demonstrate that the system maintains good dynamic and thermal stability, as evidenced by the absence of imaginary phonon modes and small lattice fluctuation at a higher temperature of 600 K. The hybrid functional calculations reveal that the monolayer exhibits a relatively small indirect gap of 1.22 eV, and the energy bands near the conduction band minimum exhibit double degeneracy with weak dispersions, which is very beneficial for enhancing the *n*-type power factor. Meanwhile, a relatively lower lattice thermal conductivity is found due to strong lattice anharmonicity caused by the antibonding state and the symmetry breaking of the structure. Collectively, a larger *ZT* value of 3.9 at 600 K can be realized for the *n*-type Janus monolayer ZrBrI at an optimal concentration of $1.89\times 10^{13}\;{\mathrm{cm}}^{-2}$, highlighting its promising thermoelectric application in the intermediate temperature region.

## 1. Introduction

Due to the increasingly severe energy crisis and environmental pollution, the development of renewable energy technologies has attracted growing attention. Among them, thermoelectric (TE) materials, which can directly convert heat into electricity, are considered promising candidates to address these challenges. It is well known that the TE conversion efficiency of a system at temperature *T* is quantified by the dimensionless figure-of-merit (*ZT*), which is defined as $ZT=S^{2}\sigma T/(\kappa_{e}+\kappa_{l})$. Here, S, σ, $\kappa_{e}$, and $\kappa_{l}$ denote the Seebeck coefficient, the electrical conductivity, the electronic thermal conductivity, and the lattice thermal conductivity, respectively. For practical and economically competitive waste heat recovery applications, a *ZT* exceeding 3.0 is required (the target set by the U.S. Department of Energy), which remains unattainable for most TE materials, thereby limiting their large-scale applications. Over the past decades, a variety of optimizing strategies or design principles have been proposed to improve TE performance, such as phonon-glass electron-crystal, low-dimensionalization, formation of superlattice, energy filtering, band convergence, resonant levels, ionic gelation, and high-entropy alloying [1,2,3,4,5,6,7,8,9,10,11,12]. It is noteworthy that the pioneering study of Hicks and Dresselhaus demonstrated that leveraging low-dimensional structures can simultaneously enhance the power factor (PF = $S^{2}\sigma$) and decrease the thermal conductivity, leading to substantially increased *ZT* values [1,2].

Over the past decades, two-dimensional (2D) materials have emerged as a focus of research interest among the global physics and materials science community [13,14,15,16,17]. For instance, Bahuguna et al. demonstrated that the GaSe monolayer simultaneously possesses a large Seebeck coefficient and electrical conductivity, which makes it an efficient candidate for low-temperature TE conversion [18]. Using first-principles calculations, Huang et al. reported the existence of the monolayer zirconium dihalide ZrX_2_ (X = Cl, Br, and I) and discussed the outstanding optoelectronic properties of the system [19]. Due to the lack of both the in-plane inversion and out-of-plane mirror symmetry, the Janus monolayers have recently attracted particular attention. In 2017, Lu et al. successfully synthesized the Janus MoSSe from the prototypical transition metal dichalcogenide (TMD) MoS_2_ via a modified chemical vapor deposition (CVD) technique, in which the top-layer S atoms were selectively substituted by Se atoms [20]. On the theoretical side, Yang et al. investigated the elastic properties of the Janus monolayer ZrBrI and confirmed its mechanical stability by density functional theory (DFT) calculations [21]. Notably, the Computational 2D Materials Database (C2DB) [22,23] reports that ZrBrI exhibits a moderate band gap of 1.17 eV, which may effectively suppress the bipolar effect and possibly enhance the electronic transport properties. By solving the phonon Boltzmann transport equation, Singh et al. predicted the room-temperature lattice thermal conductivity of zirconium halides, which are calculated to be 37.72 W/mK, 26.10 W/mK, and 46.05 W/mK for the ZrBrCl, ZrBrF, and ZrClF, respectively (with respect to effective thickness of 7.23 Å, 6.61 Å, 6.44 Å) [24]. These systems exhibit relatively high thermal conductivity, which is generally unfavorable for achieving good TE performance. However, by substituting isovalent elements such as I atoms, the phonons of the system become softened, and this consequently leads to reduced lattice thermal conductivity. Up to now, the phonon and electronic transport properties of ZrBrI have not been comprehensively investigated, despite its promising potential as a TE material due to the unique crystal structure.

In this work, first-principles calculations are combined with Boltzmann transport theory to explore the structural, electronic, and TE properties of the Janus monolayer ZrBrI. We reveal that the *n*-type ZrBrI system possesses an enhanced PF, which can be primarily attributed to the presence of flat and degenerate conduction bands near the Fermi level. Moreover, the antibonding states and the symmetry breaking of the structure induce strong anharmonicity, which significantly reduces the lattice thermal conductivity. Consequently, an optimized *ZT* of ZrBrI reaches a high value of 1.9 at 300 K and further increases to 3.9 at 600 K for the *n*-type system. Such large *ZT* values already surpass those of most previously reported 2D TE materials, highlighting its good performance in the relevant temperature window [25,26,27,28,29,30,31,32,33].

## 2. Computational Methods

The workflow for calculating the TE properties of the Janus monolayer ZrBrI is illustrated in Figure S1 of the Supplementary Material. The electronic structure is predicted by using the plane-wave pseudopotential method, as coded in the Vienna Ab initio Simulation Package (VASP) [34]. Beyond the usual Perdew–Burke–Ernzerhof (PBE) formulation for the exchange-correlation functional, we adopt the Heyd–Scuseria–Ernzerhof (HSE) hybrid functional [35], and the mixing parameter (AEXX) is set to 0.2. The plane-wave cutoff energy is 280 eV, and the Brillouin zone is sampled using a uniform 7 × 7 × 1 Monkhorst-Pack **k**-mesh. To remove interactions between the monolayer and its periodic images, a vacuum thickness of 18.23 Å is introduced along the out-of-plane direction. As the system contains heavy atoms, spin–orbit coupling (SOC) is explicitly considered in our calculations. In addition, the electronic transport coefficients (S, σ, and $\kappa_{e}$) are calculated by solving the Boltzmann transport equation [36], where the doping is treated within the rigid-band picture, and the chemical potential (μ) is employed to characterize the doping level. That is, the *n*-type doping corresponds to an upward shift of the Fermi level and positive μ, while the *p*-type doping means a downward shift of the Fermi level and thus negative μ. It should be noted that our approach goes beyond the conventional deformation potential (DP) theory, where the carrier relaxation time is often treated as a constant. In contrast, we adopt the energy-dependent carrier relaxation time, which is evaluated using the constant electron–phonon coupling approximation (CEPCA) in the TransOpt code [37]. Such an approach retains the band-structure dependence of the scattering phase space while assuming a k-independent electron–phonon coupling strength. Specifically, the carrier relaxation time can be expressed as $\tau_{nk}^{-1}=\frac{2\pi k_{B}TE_{\mathrm{def}}^{2}}{VG\hbar }\sum_{mk\prime }\delta (\varepsilon_{nk}-\varepsilon_{mk\prime })$, where the electronic band structure $\varepsilon_{nk}$, the deformation potential constant $E_{\mathrm{def}}$, and the Young’s modulus G can be obtained from first-principles calculations. The CEPCA effectively captures the variation of relaxation time arising from the electronic density of states and band dispersion, thereby significantly improving the prediction accuracy of transport coefficients compared with the commonly used constant relaxation time approximation (CRTA) or simple DP theory. In fact, this approach has been widely employed in the calculations of transport coefficients for many TE materials, including 2D systems such as the monolayer VS_2_ [38], the Janus α-TeSSe [29], the PbSe/SnSe vdW heterostructure [39], and the Janus Sn2PAs [40]. It should be mentioned that the CEPCA approach may underestimate the contribution of inter-band and inter-valley scattering. In addition, it does not sufficiently distinguish between different scattering mechanisms and may neglect or weaken the effects of processes such as polar optical phonon (POP) scattering. For systems where these scattering mechanisms dominate, it may consequently overestimate the electronic transport properties. In the present work, however, we consider both acoustic and POP scattering in the calculations, while neglecting ionized impurity scattering and boundary scattering since we focus on room and higher temperature. On the other hand, the lattice thermal conductivity can be obtained by iteratively solving the phonon Boltzmann transport equation, as executed by the ShengBTE code [41]. The required second- and third-order interatomic force constants (IFCs) are calculated using the finite displacement method with a 5 × 5 × 1 supercell, which is performed via the Phonopy program [42]. Moreover, a dense 72 × 72 × 1 q-mesh is employed to ensure the convergence of the lattice thermal conductivity. The convergence tests of the above-mentioned parameters are presented in Figure S2 of the Supplementary Material.

## 3. Results and Discussion

**Figure 1:**
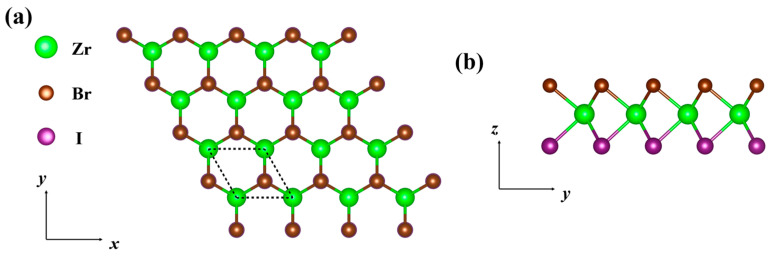
Crystal structure of the ZrBrI: (**a**) top view and (**b**) side view. The dashed lines indicate the primitive cell.

Figure 1 illustrates the crystal structure of the Janus monolayer ZrBrI. The system has a space group of *P3m1* (No. 156), and the hexagonal lattice is composed of three atoms in the primitive cell, which features a sandwich-like arrangement of Br–Zr–I. As mentioned above, the monolayer lacks both in-plane inversion symmetry and out-of-plane mirror symmetry. The optimized lattice constant is 3.703 Å, and the calculated Zr–Br and Zr–I bond lengths are respectively 2.78 Å and 2.91 Å, which agree well with those reported in the C2DB [22,23].

**Figure 2:**
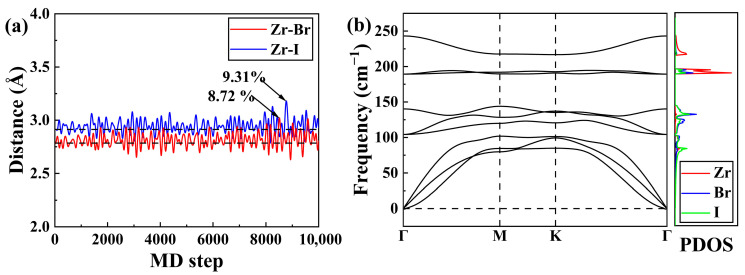
(**a**) The AIMD results (at 600 K) for the Zr–Br and Zr–I distances in the Janus ZrBrI monolayer, where the dashed lines denote the corresponding equilibrium distances. The arrows indicate the maximum fluctuation of the bond lengths from their equilibrium values. (**b**) Phonon dispersion relations and phonon density of states of the system.

The thermal stability of the Janus ZrBrI monolayer is evaluated by ab initio molecular dynamics (AIMD) simulations performed for 10,000 steps at 600 K with a time step of 1 fs, which corresponds to a total simulation time of 10 ps. Figure 2a plots the distance between the nearest-neighbor Zr–Br and Zr–I as a function of molecular dynamics (MD) step, with the dashed lines representing their respective equilibrium distances. In general, when the atomic vibration amplitude exceeds 10% of the interatomic spacing, the long-range order of the crystal cannot be maintained, and the system becomes unstable [43]. We see that the maximum fluctuation of Zr–Br (Zr–I) distance is only 8.72% (9.31%) at 600 K. Moreover, the maximum energy fluctuation is less than 5%, and the overall integrality of the crystal structure remains preserved (Figures S3 and S4 of the Supplementary Material). All these findings confirm that the Janus monolayer is thermally stable at 600 K. Furthermore, we see from Figure 2b that the phonon dispersion relations exhibit positive frequencies, confirming the dynamic stability of the monolayer.

**Figure 3:**
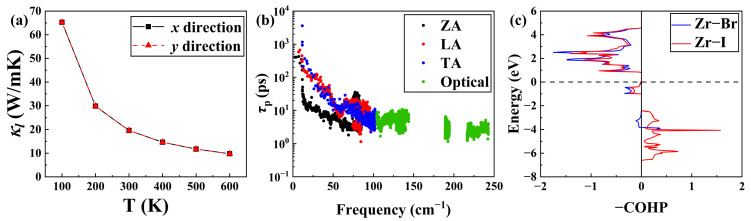
(**a**) The temperature-dependent lattice thermal conductivity of the Janus ZrBrI monolayer. (**b**) Relaxation time as a function of phonon frequency. (**c**) The corresponding COHP analysis of the nearest-neighbor atomic pairs.

We next focus on the phonon transport of the Janus ZrBrI monolayer. As can be observed from Figure 2b, the maximum phonon frequency is smaller than 250 cm^−1^, which suggests a relatively lower Debye temperature. Moreover, all the optical branches of the system exhibit very weak dispersions, indicating that the lattice thermal conductivity should be mainly contributed by the acoustic phonons. Analysis of the projected phonon density of states (PDOS) indicates that the heavier I atoms dominate the vibrations in the low-frequency region (0~100 cm^−1^). In contrast, the intermediate-frequency range (100~180 cm^−1^) is almost entirely contributed by the I and Br atoms, and the high-frequency optical modes (>180 cm^−1^) are largely associated with the vibrations of Zr atoms. Such distinct atomic contributions at different frequency ranges provide an effective way to manipulate the phonon transport behavior [44]. In addition, we see from Figure 3a that the lattice thermal conductivity of the ZrBrI monolayer is identical along the *x*- and *y*-directions. It should be mentioned that the lattice thermal conductivity of 2D materials depends on the definition of “thickness”. In this work, we adopt the sum of the buckling distance of ZrBrI (3.77 Å) and the van der Waals radii of outer atoms (2.22 Å for Br, and 2.36 Å for I), giving an effective thickness of 8.35 Å. Note that the room temperature lattice thermal conductivity of the Janus monolayer is 19.62 W/mK, which is obviously lower than those of Janus monolayer MoSSe (27.15 W/mK) [45], WSSe (47.90 W/mK) [46], and PtSTe (30.81 W/mK) [47] but somewhat higher than that of SnSSe (6.64 W/mK) [48] (all calculated with respect to effective thickness). The relatively lower $\kappa_{l}$ of the ZrBrI is beneficial for enhancing its TE performance. On the other hand, our additional calculations indicate that the bipolar thermal conductivity at 600 K is only 0.005 W/mK, which is much lower than the lattice thermal conductivity and thus can be neglected in the subsequent discussion.

It is well known that the lattice thermal conductivity can be expressed as $\kappa_{l}=\frac{1}{3}C_{v}v_{g}^{2}\tau_{p}$, where $v_{g}$, $C_{v}$ and $\tau_{p}$ are the group velocity, the heat capacity, and the phonon relaxation time, respectively. To further investigate the phonon transport behavior, Figure 3b plots the calculated $\tau_{p}$ of the ZrBrI monolayer as a function of phonon frequency. We found that the average $\tau_{p}$ (20.31 ps) of the acoustic phonons is significantly larger than that of the optical phonons (4.14 ps). Here, the average $\tau_{p}$ of the acoustic phonon modes is defined as the arithmetic mean: ${\bar{\tau }}_{p}=\frac{1}{N_{\mathrm{ac}}}\sum_{\lambda \in \mathrm{acoustic}}\tau_{\lambda }$, where $N_{\mathrm{ac}}$ is the total number of acoustic phonon modes, and $\tau_{\lambda }$ is the relaxation time of phonon mode λ. Such a difference illustrates that $\kappa_{l}$ is mainly contributed by the low-frequency acoustic phonons, which is thus the focus in the following discussion. It should be noted that the average $\tau_{p}$ is smaller than that of some Janus materials with low lattice thermal conductivity, such as SnSSe (33.56 ps) [49], indicating there is strong anharmonic scattering in the ZrBrI monolayer. On the other hand, we find that the acoustic phonons have an average $v_{g}$ of 1.25 km/s, which is relatively smaller than those of good TE materials SnSe (1.7 km/s) and PbTe (1.8 km/s) at 300 K [50,51]). In principle, the lower average $\tau_{p}$ and $v_{g}$ can be attributed to the symmetry breaking of the Janus structure, which will open the scattering channel that is originally limited by the symmetry selection rules of phonon scattering [52]. Indeed, we see from Figure S5 of the Supplementary Material that the ZrBrI monolayer exhibits relatively large three-phonon scattering phase space and Grüneisen parameters. As a result, phonon scattering can be more likely to occur, leading to small $\tau_{p}$ and $v_{g}$, which in turn reduce the lattice thermal conductivity.

To gain a deep insight into the chemical bonding characteristics of the Janus monolayer ZrBrI, the crystal orbital Hamilton population (COHP) analysis is carried out using the so-called LOBSTER code [53,54,55,56], which is based on the converged DFT results. The code is used to yield the coefficients of linear combinations of atomic orbitals (LCAOs) that, together with the overlap and Hamiltonian matrices, allow the calculations of various chemical-bonding quantities [55]. In the present calculations, the projection basis is carefully chosen to accurately describe the valence and near-Fermi-level electronic states. Specifically, the 4*s*, 4*p*, 5*s*, and 4*d* orbitals are included for the Zr atoms, the 4*s* and 4*p* orbitals for the Br atoms, and the 5*s* and 5*p* orbitals for the I atoms. It is well known that the COHP of various nearest-neighbor atomic pairs reveals the corresponding bonding strength, where the positive and negative values of −COHP correspond to the bonding and antibonding states, respectively. As illustrated in Figure 3c, there are some obvious antibonding states in a narrow energy window (−1~0 eV) below the Fermi level for both the Zr-Br and Zr-I bonds. On the other hand, the integrated COHP (namely, ICOHP) is calculated to be −1.23 and −1.29 for the Zr-Br and Zr-I bond, respectively. Although the values do not differ significantly, the presence of antibonding states below the Fermi level still weakens the bonding strength, thereby enhancing the lattice anharmonicity and resulting in a lower lattice thermal conductivity [57,58].

**Figure 4:**
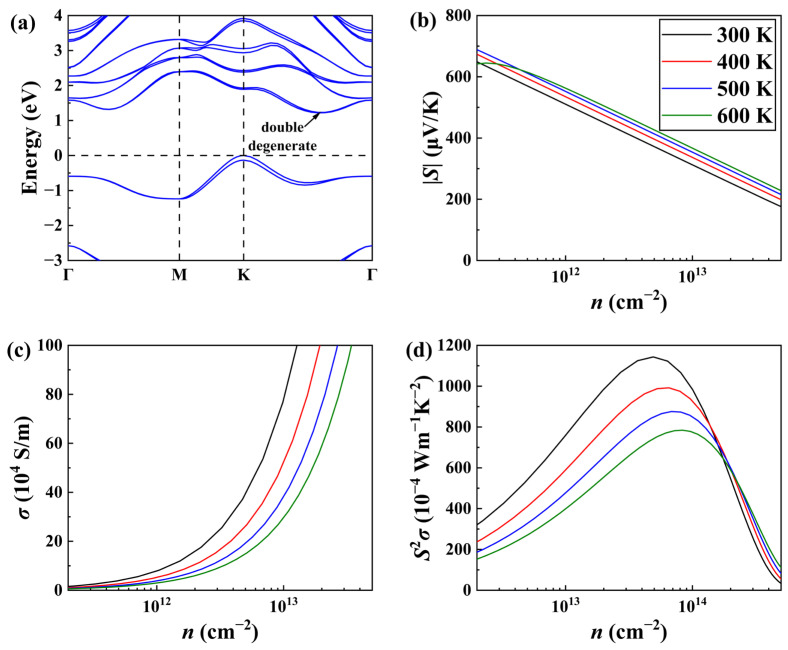
(**a**) Energy band structure of the Janus ZrBrI. (**b**) Calculated Seebeck coefficient (absolute value), (**c**) electrical conductivity, and (**d**) power factor, plotted as a function of electron concentration at different temperatures.

Figure 4a shows the energy band structure of the Janus ZrBrI monolayer obtained from HSE hybrid functional calculations, as mentioned above. We see that the system exhibits an indirect band gap of 1.22 eV, which is in good agreement with the previously reported result of 1.17 eV [22,23]. The conduction band minimum (CBM) is located between the Γ and K points, while the valence band maximum (VBM) appears at the K point. It should be noted that, for a non-centrosymmetric system, the inclusion of SOC usually lifts the band degeneracy and reduces the band gap, as compared with that shown in Figure S6 of Supplementary Material. Notably, the conduction bands close to the CBM exhibit double degeneracy and relatively weak dispersions, indicating a large effective mass of electrons and thus a potentially higher *n*-type PF.

The carrier relaxation time of the Janus ZrBrI can be obtained from the band structure by fully considering phonon scattering. To accurately calculate the deformation potential constant, the vacuum level is adopted as the reference level. Along the *x*- (*y*-) direction, the DP constants of the CBM and VBM are calculated to be 0.40 (1.18) and 1.45 (1.65) eV, respectively. In addition, the corresponding room-temperature relaxation times are 549 (161) and 204 (167) fs. This difference may be related to the band structure anisotropy of the ZrBrI and the phonon scattering in different directions. Importantly, the carrier relaxation time of the *n*-type system is much longer than that of the *p*-type along the *x*-direction, which is very beneficial to improve the electronic transport properties. Therefore, our subsequent analysis is primarily focused on the *n*-type system along the *x*-direction. By incorporating the calculated carrier relaxation time into the Boltzmann transport equation, we are now able to calculate the electronic transport coefficients of *n*-type ZrBrI, as illustrated in Figure 4b–d along the *x*-direction. For the Seebeck coefficient, Figure 4b shows the absolute value along the *x*-direction, plotted as a function of the electron concentration. At a representative carrier concentration of 1.8 × 10^12^ cm^−2^, the room temperature Seebeck coefficient exceeds 450 μV/K, which is comparable with those of several well-established TE materials, such as Bi_2_Te_3_ (~230 μV/K) [59], Mg_3_Sb_2_ (~200 μV/K) [60], and SnSe (~180 μV/K) [61,62], as well as Janus MoSSe (~100 μV/K) [63]. Of course, there may exist some differences in dimensionality and measurement conditions when making such comparisons. Similar results can be found in the temperature range from 400 to 600 K. As discussed above, the large Seebeck coefficients observed in the ZrBrI monolayer can be ascribed to the presence of doubly degenerate flat bands near the CBM. In the case of the electrical conductivity, we observe from Figure 4c that it exhibits obvious temperature dependence and shows a monotonic increase with the carrier concentration. On the other hand, the variation of the electronic thermal conductivity should be similar to that of the electrical conductivity, since they are rationalized by the well-known Wiedemann–Franz law ($\kappa_{e}=L\sigma T$, where the carrier concentration-dependent Lorenz number *L* is shown in Figure S7 of the Supplementary Material). Due to the competing behavior between the S and σ, the optimized PF appears at an intermediate carrier concentration (4.91 × 10^13^ cm^−2^ ~ 8.35 × 10^13^ cm^−2^), as illustrated in Figure 4d.

**Figure 5:**
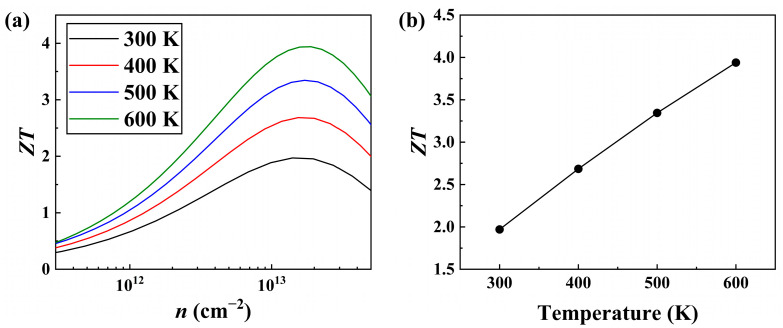
(**a**) *ZT* values of the Janus ZrBrI with respect to electron concentration at different temperatures. (**b**) Optimal *ZT* values as a function of temperature.

By combining the electronic and phonon transport coefficients, the dimensionless *ZT* for the *n*-type Janus ZrBrI monolayer can be readily obtained. In Figure 5a, we plot the *ZT* value along the *x*-direction as a function of temperature and carrier concentration, where the optimal value can be achieved by fine-tuning the concentration. In particular, at an optimized electron concentration of 1.40 × 10^13^ cm^−2^, a maximum *ZT* of 1.9 can be obtained at 300 K, which is comparable with that of the benchmark TE material Bi_2_Te_3_ [64]. Meanwhile, compared with most previously reported 2D systems such as In_2_SeO (0.23) [26], SnSSe (0.28) [30], and PdSTe (0.68) [31], our Janus ZrBrI exhibits obviously higher *ZT* value, rendering it a promising candidate for room-temperature TE applications. Moreover, we see from Figure 5b that the *ZT* values exhibit a nearly linear increase with temperature, indicating that enhanced TE performance may be realized in the ZrBrI monolayer if it is operated in a higher temperature region. In particular, a maximum *ZT* value of 3.9 can be reached at 600 K, with an optimized electron concentration of 1.80 × 10^13^ cm^−2^. Such state-of-the-art TE performance is superior to that of many reported 2D materials, including the HfSSe (0.81) [28] and the TeSSe (1.95) [29]. All the above results collectively highlight that ZrBrI could be a highly promising candidate for intermediate-temperature TE applications, and much experimental effort should be devoted to synthesizing such halogen-based monolayer systems using possible techniques such as modified CVD [20] by overcoming challenges such as the high volatility of halogen elements and their relatively poor chemical stability, as well as considering substrate compatibility or device-level applicability.

## 4. Summary

In summary, our first-principles calculations indicate that the Janus ZrBrI monolayer is both thermally and dynamically stable and exhibits a low lattice thermal conductivity caused by its pronounced lattice anharmonicity, originating from the presence of antibonding states below the Fermi level and breaking both inversion and mirror symmetries. In addition, a remarkably high *n*-type power factor is found, as governed by the doubly degenerate flat conduction bands in the vicinity of the Fermi level. Consequently, a room-temperature *ZT* of 1.9 and an even higher value of 3.9 at 600 K can be realized for the system. It is worth mentioning that, owing to the presence of relatively cheap halogen elements in its composition, ZrBrI exhibits a lower cost compared with the conventional bulk TE material, such as Bi_2_Te_3_, as well as the experimentally synthesized Janus monolayer MoSSe.

## Data Availability

The original contributions presented in this study are included in the article/Supplementary Material. Further inquiries can be directed to the corresponding author.

## Supplementary Materials

The following supporting information can be downloaded at: https://www.mdpi.com/article/10.3390/ma19091716/s1, Figure S1: Computational workflow used to obtain the thermoelectric transport properties. Figure S2: Convergence tests of (a) cutoff energy, (b) *k*-mesh, and (c) *q*-mesh. Figure S3: The AIMD result of the total energy at 600 K, where the red dashed line denotes the minimum energy and the arrow shows the maximum energy fluctuation. Figure S4: Snapshot of the atomic configurations during the AIMD simulations at 600 K. (a,b) correspond to the top- and side- view at the maximum fluctuation (9.31%) of the Zr–I bond length, respectively. (c,d) refer to those at the maximum fluctuation (8.72%) of the Zr–Br bond length. Figure S5: (a) The Grüneisen parameter and (b) the three-phonon scattering phase space of Janus ZrBrI, plotted with respect to the phonon frequency. Figure S6: The electronic band structure of Janus ZrBrI without consideration of SOC. Figure S7: Lorenz number of the Janus ZrBrI as a function of electron concentration at different temperatures.

## References

[1] Hicks L.D., Dresselhaus M.S. (1993). Effect of quantum-well structures on the thermoelectric figure of merit. Phys. Rev. B.

[2] Hicks L.D., Dresselhaus M.S. (1993). Thermoelectric figure of merit of a one-dimensional conductor. Phys. Rev. B.

[3] Rowe D.M. (1995). CRC Handbook of Thermoelectric.

[4] Venkatasubramanian R., Siivola E., Colpitts T., O’Quinn B. (2001). Thin-film thermoelectric devices with high room-temperature figures of merit. Nature.

[5] Snyder G.J., Christensen M., Nishibori E., Caillat T., Iversen B.B. (2004). Disordered zinc in Zn4Sb3 with phonon-glass and electron-crystal thermoelectric properties. Nat. Mater..

[6] Heremans J.P., Thrush C.M., Morelli D.T. (2004). Thermopower enhancement in lead telluride nanostructures. Phys. Rev. B.

[7] Faleev S.V., Léonard F. (2008). Theory of enhancement of thermoelectric properties of materials with nanoinclusions. Phys. Rev. B.

[8] Heremans J.P., Jovovic V., Toberer E.S., Saramat A., Kurosaki K., Charoenphakdee A., Yamanaka S., Snyder G.J. (2008). Enhancement of Thermoelectric Efficiency in PbTe by Distortion of the Electronic Density of States. Science.

[9] Pei Y., Shi X., LaLonde A., Wang H., Chen L., Snyder G.J. (2011). Convergence of electronic bands for high performance bulk thermoelectrics. Nature.

[10] Biswas K., He J., Blum I.D., Wu C.-I., Hogan T.P., Seidman D.N., Dravid V.P., Kanatzidis M.G. (2012). High-performance bulk thermoelectrics with all-scale hierarchical architectures. Nature.

[11] He J., Kanatzidis M.G., Dravid V.P. (2013). High performance bulk thermoelectrics via a panoscopic approach. Mater. Today.

[12] Han C.-G., Qian X., Li Q., Deng B., Zhu Y., Han Z., Zhang W., Wang W., Feng S.-P., Chen G. (2020). Giant thermopower of ionic gelatin near room temperature. Science.

[13] Novoselov K.S., Geim A.K., Morozov S.V., Jiang D., Zhang Y., Dubonos S.V., Grigorieva I.V., Firsov A.A. (2004). Electric Field Effect in Atomically Thin Carbon Films. Science.

[14] Novoselov K.S., Jiang D., Schedin F., Booth T.J., Khotkevich V.V., Morozov S.V., Geim A.K. (2005). Two-dimensional atomic crystals. Proc. Natl. Acad. Sci. USA.

[15] Naguib M., Kurtoglu M., Presser V., Lu J., Niu J., Heon M., Hultman L., Gogotsi Y., Barsoum M.W. (2011). Two-Dimensional Nanocrystals Produced by Exfoliation of Ti3AlC2. Adv. Mater..

[16] Liu G.-B., Xiao D., Yao Y., Xu X., Yao W. (2015). Electronic structures and theoretical modelling of two-dimensional group-VIB transition metal dichalcogenides. Chem. Soc. Rev..

[17] Yi Y., Chen Z., Yu X., Zhou Z., Li J. (2019). Recent Advances in Quantum Effects of 2D Materials. Adv. Quantum Technol..

[18] Bahuguna B.P., Saini L.K., Sharma R.O., Tiwari B. (2018). Hybrid functional calculations of electronic and thermoelectric properties of GaS, GaSe, and GaTe monolayers. Phys. Chem. Chem. Phys..

[19] Huang X., Zhuo Z., Yan L., Wang Y., Xu N., Song H.-Z., Zhou L. (2021). Single-Layer Zirconium Dihalides ZrX2 (X = Cl, Br, and I) with Abnormal Ferroelastic Behavior and Strong Anisotropic Light Absorption Ability. J. Phys. Chem. Lett..

[20] Lu A.-Y., Zhu H., Xiao J., Chuu C.-P., Han Y., Chiu M.-H., Cheng C.-C., Wei K.-H., Yang Y., Wang Y. (2017). Janus monolayers of transition metal dichalcogenides. Nat. Nanotechnol..

[21] Yang M., Chen L., Huang D., Huang X. (2024). First principles study on the elastic properties of two-dimensional Janus ZrXY (X/Y = Cl, Br, and I, X ≠ Y). AIP Adv..

[22] Haastrup S., Strange M., Pandey M., Deilmann T., Schmidt P.S., Hinsche N.F., Gjerding M.N., Torelli D., Larsen P.M., Riis-Jensen A.C. (2018). The Computational 2D Materials Database: High-throughput modeling and discovery of atomically thin crystals. 2D Mater..

[23] Gjerding M.N., Taghizadeh A., Rasmussen A., Ali S., Bertoldo F., Deilmann T., Knøsgaard N.R., Kruse M., Larsen A.H., Manti S. (2021). Recent progress of the Computational 2D Materials Database (C2DB). 2D Mater..

[24] Singh J., Singh G., Tripathi S.K. (2023). Janus zirconium halide ZrXY (X, Y = Br, Cl and F) monolayers with high lattice thermal conductivity and strong visible-light absorption. Phys. Chem. Chem. Phys..

[25] Patel A., Singh D., Sonvane Y., Thakor P.B., Ahuja R. (2020). High Thermoelectric Performance in Two-Dimensional Janus Monolayer Material WS-X (X = Se and Te). ACS Appl. Mater. Interfaces.

[26] Vu T.V., Nguyen C.V., Phuc H.V., Lavrentyev A.A., Khyzhun O.Y., Hieu N.V., Obeid M.M., Rai D.P., Tong H.D., Hieu N.N. (2021). Theoretical prediction of electronic, transport, optical, and thermoelectric properties of Janus monolayers In_2_XO (X = S, Se, Te). Phys. Rev. B.

[27] Vu T.V., Vi V.T.T., Phuc H.V., Nguyen C.V., Poklonski N.A., Duque C.A., Rai D.P., Hoi B.D., Hieu N.N. (2021). Electronic, optical, and thermoelectric properties of janus in-based monochalcogenides. J. Phys. Condens. Matter.

[28] Bera J., Betal A., Sahu S. (2021). Spin orbit coupling induced enhancement of thermoelectric performance of HfX_2_ (X = S, Se) and its Janus monolayer. J. Alloys Compd..

[29] Chen S., Chen X., Zeng Z., Geng H., Yin H. (2021). The coexistence of superior intrinsic piezoelectricity and thermoelectricity in two-dimensional janus α-TeSSe. Phys. Chem. Chem. Phys..

[30] Bai S., Tang S., Wu M., Luo D., Zhang J., Wan D., Yang S. (2022). Unravelling the thermoelectric properties and suppression of bipolar effect under strain engineering for the asymmetric janus SnSSe and PbSSe monolayers. Appl. Surf. Sci..

[31] Jakhar M., Sharma R., Kumar A. (2023). Janus β-PdXY (X/Y = S, Se, Te) materials with high anisotropic thermoelectric performance. Nanoscale.

[32] Chauhan P., Singh J., Kumar A. (2023). As-based ternary Janus monolayers for efficient thermoelectric and photocatalytic applications. J. Mater. Chem. A.

[33] Wu Y.-L., Yang Q., Geng H.-Y., Cheng Y. (2024). The thermoelectric properties of CdBr, CdI, and Janus Cd2BrI monolayers with low lattice thermal conductivity. Phys. Chem. Chem. Phys..

[34] Kresse G., Furthmüller J. (1996). Efficient iterative schemes for ab initio total-energy calculations using a plane-wave basis set. Phys. Rev. B.

[35] Heyd J., Scuseria G.E., Ernzerhof M. (2003). Hybrid functionals based on a screened Coulomb potential. J. Chem. Phys..

[36] Madsen G.K.H., Singh D.J. (2006). BoltzTraP. A code for calculating band-structure dependent quantities. Comput. Phys. Commun..

[37] Li X., Zhang Z., Xi J., Singh D.J., Sheng Y., Yang J., Zhang W. (2021). TransOpt. A code to solve electrical transport properties of semiconductors in constant electron–phonon coupling approximation. Comput. Mater. Sci..

[38] Cui Y., Fan W., Liu X., Ren J., Gao Y. (2021). Electronic conductivity of two-dimensional VS2 monolayers: A first principles study. Comput. Mater. Sci..

[39] Tang S., Ai P., Bai S., Wan D., Li X., Guo W., Zheng T., Wang H. (2024). Weak interatomic interactions induced low lattice thermal conductivity in 2D/2D PbSe/SnSe vdW heterostructure. Mater. Today Phys..

[40] Xu Z., Gao G. (2025). Enhanced thermoelectric performance of janus Sn2PAs monolayer compared with its parents of SnP and SnAs. 2D Mater..

[41] Li W., Carrete J., Katcho N.A., Mingo N. (2014). ShengBTE: A solver of the Boltzmann transport equation for phonons. Comput. Phys. Commun..

[42] Togo A., Tanaka I. (2015). First principles phonon calculations in materials science. Scr. Mater..

[43] Goldoni G., Peeters F.M. (1996). Stability, dynamical properties, and melting of a classical bilayer Wigner crystal. Phys. Rev. B.

[44] Lu S., Ren W., He J., Yu C., Jiang P., Chen J. (2022). Enhancement of the lattice thermal conductivity of two-dimensional functionalized MXenes by inversion symmetry breaking. Phys. Rev. B.

[45] Wei D., Zhou E., Zheng X., Wang H., Shen C., Zhang H., Qin Z., Qin G. (2022). Electric-controlled tunable thermal switch based on Janus monolayer MoSSe. npj Comput. Mater..

[46] Qin H., Ren K., Zhang G., Dai Y., Zhang G. (2022). Lattice thermal conductivity of Janus MoSSe and WSSe monolayers. Phys. Chem. Chem. Phys..

[47] Pan L., Wang Z., Carrete J., Madsen G.K.H. (2022). Thermoelectric properties of the Janus PtSTe monolayer compared with its parent structures. Phys. Rev. Mater..

[48] Gupta R., Dongre B., Bera C., Carrete J. (2020). The Effect of Janus Asymmetry on Thermal Transport in SnSSe. J. Phys. Chem. C.

[49] Luo Y., Han S., Hu R., Yuan H., Jiao W., Liu H. (2021). The Thermal Stability of Janus Monolayers SnXY (X, Y = O, S, Se): Ab-Initio Molecular Dynamics and Beyond. Nanomaterials.

[50] Zhao L.-D., Lo S.-H., Zhang Y., Sun H., Tan G., Uher C., Wolverton C., Dravid V.P., Kanatzidis M.G. (2014). Ultralow thermal conductivity and high thermoelectric figure of merit in SnSe crystals. Nature.

[51] Xiao Y., Chang C., Pei Y., Wu D., Peng K., Zhou X., Gong S., He J., Zhang Y., Zeng Z. (2016). Origin of low thermal conductivity in SnSe. Phys. Rev. B.

[52] Lindsay L., Broido D.A., Mingo N. (2010). Flexural phonons and thermal transport in graphene. Phys. Rev. B.

[53] Dronskowski R., Bloechl P.E. (1993). Crystal orbital hamilton populations (COHP): Energy-resolved visualization of chemical bonding in solids based on density-functional calculations. J. Phys. Chem..

[54] Maintz S., Esser M., Dronskowski R. (2016). Efficient rotation of local basis functions using real spherical harmonics. Acta Phys. Pol. B.

[55] Maintz S., Deringer V.L., Tchougreeff A.L., Dronskowski R. (2016). LOBSTER: A tool to extract chemical bonding from plane-wave based DFT. J. Comput. Chem..

[56] Nelson R., Ertural C., George J., Deringer V.L., Hautier G., Dronskowski R. (2020). LOBSTER: Local orbital projections, atomic charges, and chemical-bonding analysis from projector-augmented-wave-based density-functional theory. J. Comput. Chem..

[57] Tang S., Zheng T., Wan D., Li X., Yan T., Guo W., Wang H., Qi X., Bai S. (2025). Low lattice thermal conductivity induced by antibonding sp-hybridization in Sb2Sn2Te6 monolayer with high thermoelectric performance: A First-principles calculation. Colloids Surf. A Physicochem. Eng. Asp..

[58] Minhas H., Sharma R.K., Pathak B. (2025). Antibonding States Drive Anharmonicity and Low Thermal Conductivity in Edge-Sharing Metal Chalcogenides. ACS Appl. Mater. Interfaces.

[59] Jeon H.W., Ha H.P., Hyun D.B., Shim J.D. (1991). Electrical and Thermoelectrical Properties of Undoped Bi_2_Te_3_-Sb_2_Te_3_ and Bi_2_Te_3_Sb_2_Te_3_-Sb_2_Se_3_ Single Crystals. J. Phys. Chem. Solids.

[60] Imasato K., Fu C., Pan Y., Wood M., Kuo J.J., Felser C., Snyder G.J. (2020). Metallic n-type Mg_3_Sb_2_ single crystals demonstrate the absence of ionized impurity scattering and enhanced thermoelectric performance. Adv. Mater..

[61] Zhao L.-D., Chang C., Tan G., Kanatzidis M.G. (2016). SnSe: A remarkable new thermoelectric material. Energy Environ. Sci..

[62] Chang C., Wu M., He D., Pei Y., Wu C.-F., Wu X., Yu H., Zhu F., Wang K., Chen Y. (2018). 3D charge and 2D phonon transports leading to high out-of-plane ZT in n-type SnSe crystals. Science.

[63] Anisha, Kumar R., Singh M., Srivastava S., Kumar T. (2025). Enhancing the thermoelectric performance of Janus MoSSe monolayer via pressure. Eur. Phys. J. Plus.

[64] Witting I.T., Chasapis T.C., Ricci F., Peters M., Heinz N.A., Hautier G., Snyder G.J. (2019). The thermoelectric properties of bismuth telluride. Adv. Electron. Mater..

