# Supplementary material for: Promising Thermoelectric Performance of Janus Monolayer ZrBrI
Source: Materials (Basel). 2026 Apr 23;19(9):1716. doi: 10.3390/ma19091716 (PMC13165224; doi:10.3390/ma19091716)
Supplement: Supplementary file 1 [file materials-19-01716-s001.zip › materials-4210732-supplementary.pdf]

Supplementary Material

Promising thermoelectric performance of Janus monolayer ZrBrI

Jingfeng Wang, Wenyan Jiao, Zihe Li, Huijun Liu*

Key Laboratory of Artificial Micro- and Nano-Structures of Ministry of Education and School of Physics and Technology, Wuhan University, Wuhan 430072, China

Contents:

1. Computational workflow

2. Convergence test

3. AIMD thermal stability

4. Phonon scattering phase space and Grüneisen parameter

5. Band structure

6. Lorenz number

* Author to whom correspondence should be addressed. Electronic mail: phlhj@whu.edu.cn

1. Computational workflow

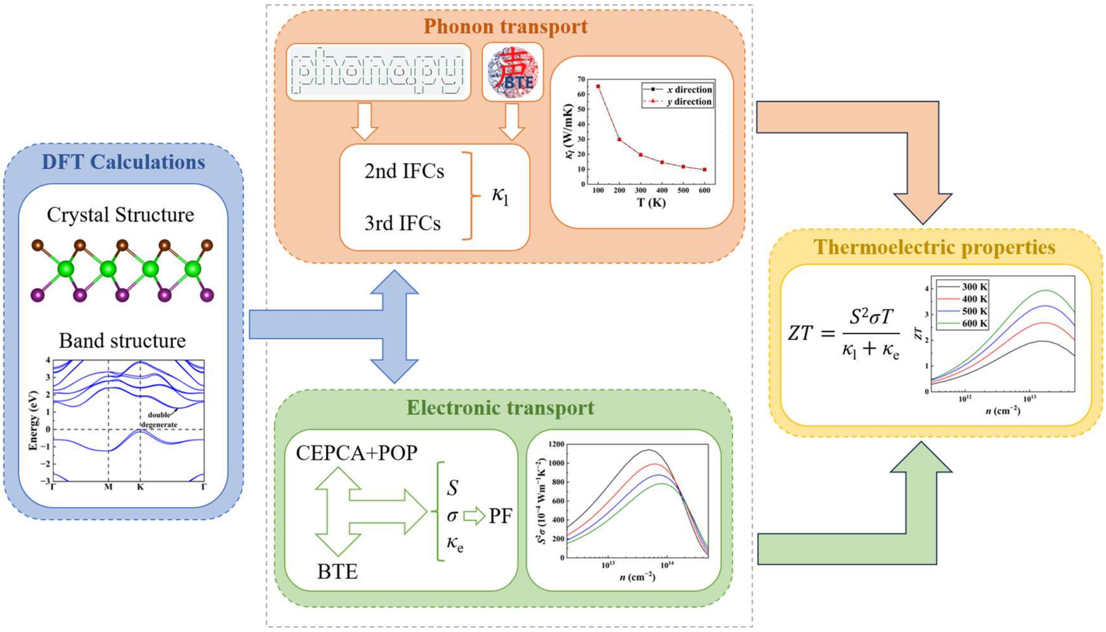

Figure S1. Computational workflow used to obtain the thermoelectric transport properties.

2. Convergence test

To ensure the reliability of our calculated results, sufficiently large cutoff energy and ***k***- and **q**- mesh should be used. As can be see from Figure S2, a plane-wave cutoff energy of 280 eV and a 7×7×1 ***k***-mesh are sufficient to ensure convergence of total energy, while a 72×72×1 ***q***-mesh is enough to reliably predict the lattice thermal conductivity. For the electronic transport coefficients, a ***k***-mesh sampling of 240/*a* (here *a* is the lattice constant) is generally assumed to obtain converged results. Consequently, we use a very dense ***k***-mesh of 91×91×1 in the calculations.

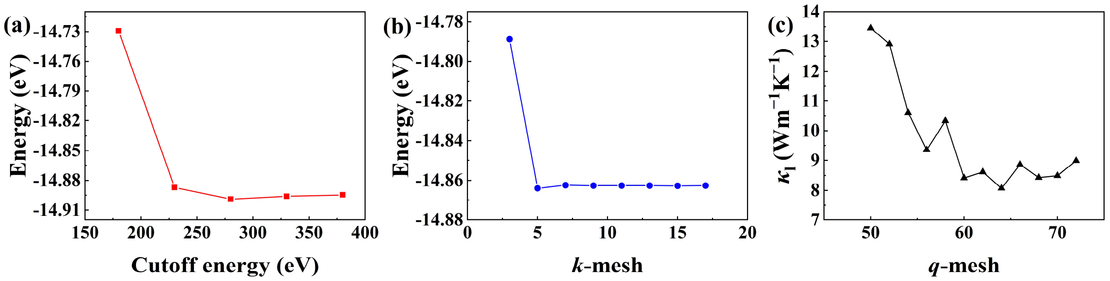

Figure S2. Convergence tests of (a) cutoff energy, (b) ***k***-mesh, and (c) ***q***-mesh.

3. AIMD thermal stability

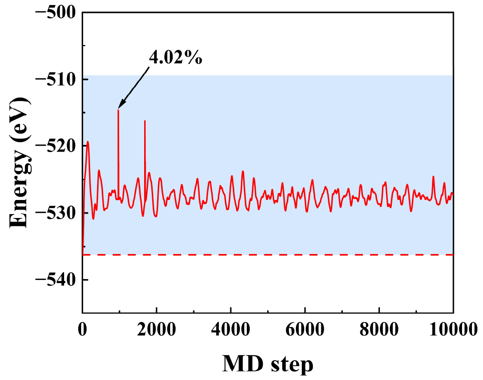

Figure S3. The AIMD result of the total energy at 600 K, where the red dashed line denotes the minimum energy and the arrow shows the maximum energy fluctuation.

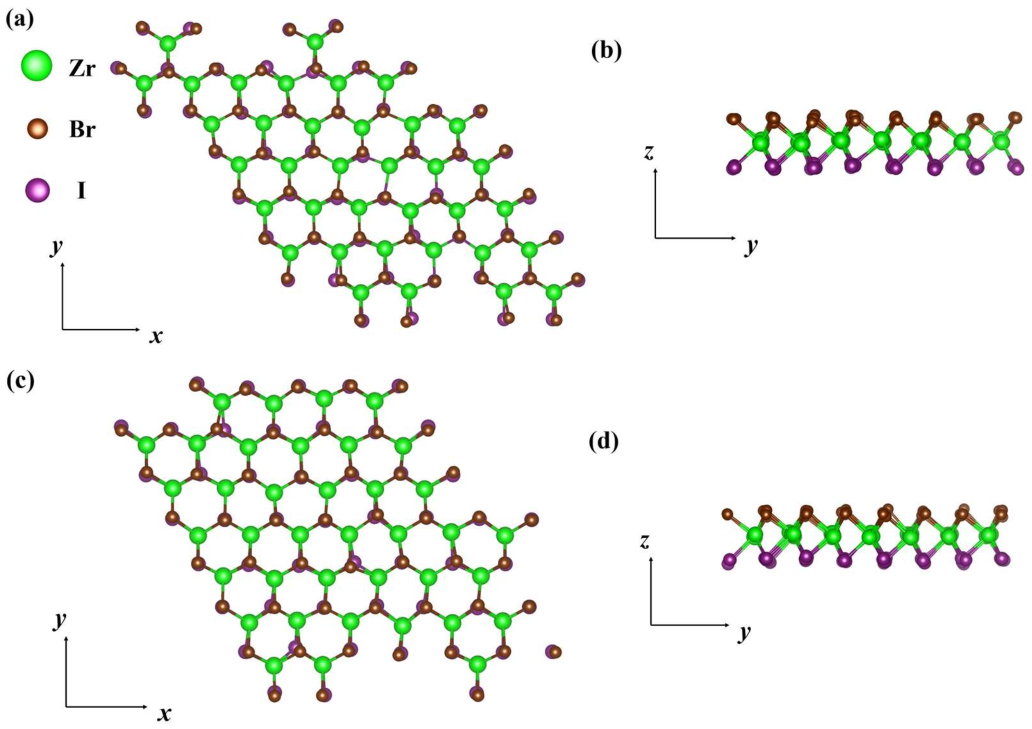

Figure S4. Snapshot of the atomic configurations during the AIMD simulations at 600 K. (a) and (b) correspond to the top- and side- view at the maximum fluctuation (9.31%) of the Zr–I bond length, respectively. (c) and (d) refer to those at the maximum fluctuation (8.72%) of the Zr–Br bond length.

4. Phonon scattering phase space and Grüneisen parameter

As shown in Figure S4, the Janus ZrBrI exhibits relatively large scattering phase space and Grüneisen parameter, which suggest its intrinsically lower lattice thermal conductivity.

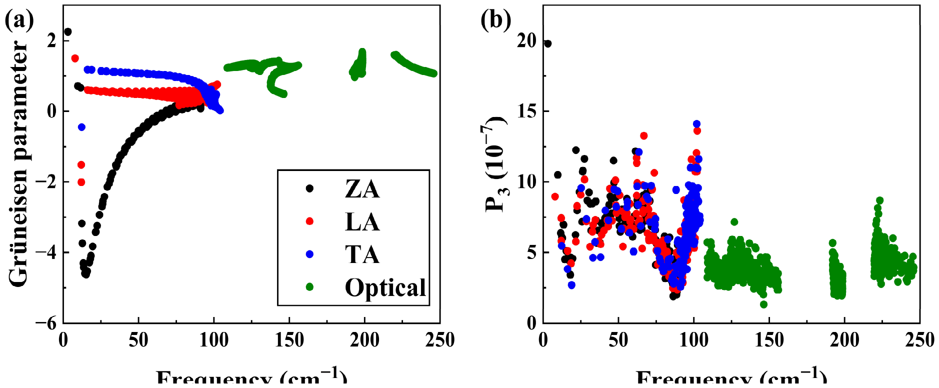

Figure S5. (a) The Grüneisen parameter and (b) the three-phonon scattering phase space of Janus ZrBrI, plotted with respect to the phonon frequency.

5. Band structure

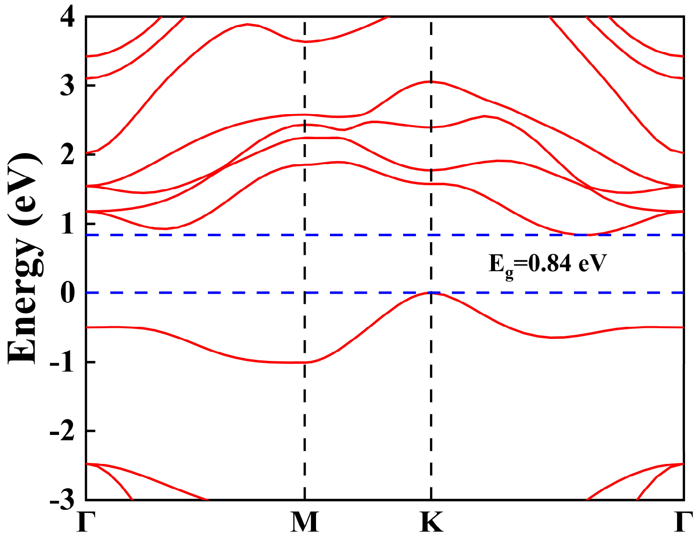

Figure S6. The electronic band structure of Janus ZrBrI without consideration of SOC.

6. Lorenz number

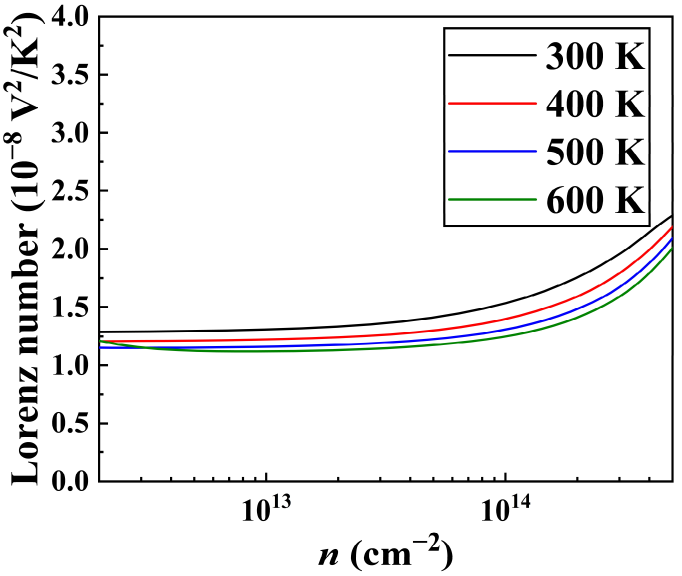

Figure S7. Lorenz number of the Janus ZrBrI as a function of electron concentration at different temperatures.
